# *Lannea acida* A. Rich. (Anacardiaceae) Ethanol Extract Exhibits Estrogenic Effects and Prevents Bone Loss in an Ovariectomized Rat Model of Osteoporosis

**DOI:** 10.1155/2017/7829059

**Published:** 2017-11-27

**Authors:** Mouchili Riepouo Oumarou, Stéphane Zingue, Berlise Yengwa Bakam, Sylvin Benjamin Ateba, Simplice Harquin Foyet, Fritz Teddy Tchaptchet Mbakop, Dieudonné Njamen

**Affiliations:** ^1^Department of Animal Biology and Physiology, Faculty of Science, University of Yaounde 1, P.O. Box 812, Yaounde, Cameroon; ^2^Department of Life and Earth Sciences, Higher Teachers' Training College, University of Maroua, P.O. Box 55, Maroua, Cameroon; ^3^Department of Biological Sciences, Faculty of Sciences, University of Maroua, P.O. Box 814, Maroua, Cameroon

## Abstract

Phytoestrogens have been shown to prevent postmenopausal osteoporosis.* Lannea acida* is a medicinal plant traditionally used in Cameroon to treat infertility, gynaecological complaints, and rheumatism. These uses prompted us to evaluate estrogenic activity of* Lannea acida* bark ethanolic extract and its antiosteoporotic potential in ovariectomized Wistar rats.* In vitro*, the E-screen assay was used to assess the ability of* L. acida* extract to induce MCF-7 cells proliferation.* In vivo*, a 3-day uterotrophic assay and a 12-week oral treatment in ovariectomized adult rats were carried out to evaluate the ability of* L. acida* extract to prevent bone mass loss.* L. acida* extract induced MCF-7 cell proliferation.* In vivo*, it significantly increased the uterine wet weight, uterine and vaginal epithelial heights, and mammary glands differentiation. At 200 mg/kg, a long-term treatment with the extract prevented body weight gain (*p* < 0.05) and loss of bone mass and/or density (*p* < 0.05) induced by ovariectomy. Also, a significant (*p* < 0.001) decrease of alkaline phosphatase activity was observed with 50 mg/kg.* L. acida* extract improved bone microarchitecture and could restore normal bone mineralization by increasing the inorganic phosphorus and calcium level in bone. These findings provide evidence that* Lannea acida* is a potential alternative for the prevention of postmenopausal osteoporosis.

## 1. Introduction

Estrogen is steroidal hormone mainly produced by ovaries, which greatly fluctuates during women's lifespan and declines with menopause [1]. In addition to its role in primary organs like uterus, vagina, and mammary glands, it plays an important role in bone growth and homeostasis. Genetic, nutritional, and lifestyle factors as well as estrogen deficiency following the menopause are known to increase osteoclastic bone resorption rate and then risk of osteoporosis [2, 3]. Osteoporosis is a worldwide threat characterized by a bone mass loss linked to a low mineral content and microarchitectural deterioration of bone tissue leading to bone fragility and increased risk of fracture [4]. These fractures occur mainly in hip, spine, and wrist and are a major cause of morbidity and mortality in elderly populations [3, 5]. According to the International Osteoporosis Foundation [6], the number of elderly people is increasing throughout the globe. Accordingly, the incidence of related fractures and costs for treatment will rise dramatically unless effective prophylactic measures are taken. It is estimated that the incidence of hip fracture will rise from 1.66 million in 1990 to 6.26 million by 2050 [7]. Osteoporosis and fragility fractures are believed to be uncommon in Africa. However, in a 2-year period study conducted in two urban hospitals in Cameroon, Zebaze and Seeman [8] reported that among all women patients aged 35 years and older diagnosed for fracture the hip and wrist fractures related to bone fragility were the most common pattern.

For decades, effective treatment such as antiresorptive bisphosphonates and hormone replacement therapy (HRT) has been used for managing postmenopausal osteoporosis [9, 10]. Unfortunately, the uses of bisphosphonates are often associated with gastrointestinal intolerance and osteonecrosis of the jaws [11]. Moreover, HRT is involved in adverse outcomes after long-term use such as increased risk of endometrial and breast cancers, stroke, and pulmonary thromboembolism [12, 13]. Due to these reports women turned to alternatives. Efforts have been made for decades to find nonhormonal, effective and safer antiosteoporotic alternatives. In line with this interest in phytoestrogens rose significantly as they mimic estrogenic activity and might be promising.

In our ongoing research of novel phytoestrogens from plant, we sought a scientific rationale for the traditional use of* Lannea acida* (syn.* Odina acida*) (Anacardiaceae), commonly called “faruhi” in Fulani-Fulfulde (Nigeria), “faàrú” in Hausa, “Mipadi” in Giziga, or “Timbiya” in Moundang in Cameroun [14], and growth in Sub-Saharan Africa. Barks of* L. acida* are traditionally used in Nigeria as antiabortifacient, vermifuge and to treat anal haemorrhoids, diarrhoea, dysentery, malnutrition, and debility [15] and in Cameroon to treat dysmenorrhea, amenorrhea, and infertility, while the leaves treat rheumatism [16]. Information provided by the traditional healer in Moutourwa (Far North Region of Cameroon) revealed that the maceration of* L. acida* stem bark in local alcoholic drink (palm wine) is used to treat diarrhoea and gynaecological complaints. Ahmed et al. [17] showed that the methanolic extract of* L. acida* increased mobility, morphology, and sperm count as well as testosterone level in Wistar rats. Moreover, 4 flavonoids have been isolated from barks of* L. acida* [18], but to the best of our knowledge, no estrogenic activity of this plant has been investigated to date. Therefore, the present study aimed to evaluate* in vitro* and* in vivo* estrogenic activities of the ethanolic extract of stem bark of* L. acida* and to assess the antiosteoporotic potential of this extract in ovariectomized Wistar rats.

## 2. Materials and Methods

### 2.1. Chemicals

17*β*-Estradiol valerate (Progynova® 2 mg) was purchased from DELPHARM (Lille, France). Penicillin (Xtapen®) was provided by CSPC Zhongnuo Pharmaceutical (Shijiazhuang City, China). Diclofenac (Dicloecnu®) was provided by ECNU Pharmaceutical (Yanzhou City, China). The cell culture medium was purchased from Cultilab (São Paulo, SP). Serum and antibiotics were purchased from GIBCO (Grand Island, NY). The 17*β*-estradiol benzoate [(estr-1,3,5(10)-trien-3,16*α*,17*β*-triol); purity ≥ 98%] was obtained from Sigma-Aldrich (Hamburg, Germany). The 2-[4-(2-hydroxyethyl)piperazin-1-yl]ethane sulfonic acid (HEPES, purity ≥ 99.5%) was purchased from Ludwig Biotecnologia Ltda (Alvorada, RS, Brazil). Trypan blue, quercetin, Alamar Blue, and Sulforhodamine-B were purchased from Sigma-Aldrich (St. Louis, MO, USA).

### 2.2. Plant Material

#### 2.2.1. Collection and Authentication

The stem barks of* Lannea acida* A. Rich. (Anacardiaceae) were harvested in Moutourwa (Far North Region of Cameroon) on 6 July 2014 (pluvial season) around 11:30 a.m. The plant was localized at the geographical coordinates of N10°12.681′ East and E0 14°11.623′ altitude with a “ESTREX” Global Positioning System. This botanical sample was identified and authenticated by Mr. Victor Nana, botanist at the National Herbarium of Cameroon (HNC-IRA), by comparison to the specimen deposited under the voucher number 40942 HNC.

#### 2.2.2. Extraction

The collected stem barks of* L. acida* were cleaned and air-dried at room temperature for 14 days. Then, 2000 g of the powder was macerated in 95% ethanol at room temperature (6 L of solvent** ×** 3, 48 h per extraction). The combined solutions were filtered using a Whatman filter paper number 4 and evaporated under reduced pressure (337 mbar at 40°C) using a rotary evaporator to afford 272 g of crude extract (a yield of 13.6%). The extract was kept at 4–8°C and dissolved in distilled water prior to administration.

#### 2.2.3. Preliminary Phytochemical Screening

Investigation on phytochemical composition of* L. acida* ethanol extract was performed according to the method described by Odebiyi and Sofowora [19]. Concentrations of some bioactive phytochemicals, such as total phenols, flavonoids, and alkaloids, were measured according to methods described by Singleton and Rossi [20], Zhishen et al. [21], and Fazel et al. [22], respectively.

### 2.3. *In Vitro* Experiments

#### 2.3.1. Cell Line System

The human ER^+^ breast adenocarcinoma MCF-7 cells were obtained from the Rio de Janeiro Cell Bank (Federal University of Rio de Janeiro, Brazil).

#### 2.3.2. Cell Culture

MCF-7 cells were cultured in RPMI-1640 medium supplemented with fetal bovine serum (FBS) 10% as previously reported by Zingue et al. [23]. Briefly, cell cultures were also supplemented with penicillin 100 U/mL, streptomycin 100 *μ*g/mL, and HEPES 10 mM. They were maintained at 37°C in a CO_2_ 5% humidified atmosphere and pH 7.4. Every two days, cells were passaged by removing 90% of the supernatant and replaced by fresh medium. Viable cells (a minimum of 95%) were checked at the beginning of the experiment by Trypan Blue dye exclusion test.

#### 2.3.3. Cell Viability Assay

Cytotoxicity potential of* L. acida* extract was evaluated by Alamar Blue (resazurin) assay in MCF-7 cells as described in our previous report [24]. This assay evaluates the mitochondrial production as a measurement of cell viability. To evaluate the influence of concentration and time on cytotoxicity, 1 × 10^4^ cells/well were seeded in a 96-well plate in 100 *μ*L of culture medium. After 24 h to permit their adhesion, cells were exposed to* L. acida* extract at concentrations from 0.1 to 200 *μ*g/mL for 24 h. The CC_50_ (cytotoxic concentration, which means concentration required to reduce the cell number by 50%) was determined by nonlinear regression analysis of the logarithm of concentration in function of the normalized response (percentage of cell viability) using the software GraphPad Prism 5.0. Each experiment was performed at least in triplicate and repeated three times.

#### 2.3.4. E-Screen Assay

In order to evaluate estrogenic-like effects of* L. acida* extract a simple and sensitive E-screen cell proliferation assay was performed with MCF-7 cells. This assay indirectly determines the estrogenicity/antiestrogenicity of compounds through measurement of the proliferation of MCF-7 cells and was performed as previously described by Zingue et al. [23]. Briefly, cells were trypsinized and seeded in 24-well plates at an initial concentration of 5 × 10^4^ cells per well in RPMI supplemented with FBS 10%. After 24 h of incubation (37°C, CO_2_ 5%) to permit their adhesion, cells were washed with phosphate-buffered saline-PBS (NaCl 137 mM; KCl 2.7 mM; Na_2_HPO_4_ 10 mM; KH_2_PO_4_ 1.8 mM; pH 7.4) and the Serum Replacement 2 (0.5x) supplemented phenol red-free RPMI was substituted for the seeding medium. A stock solution of* L. acida *ethanolic extract 100 mg/mL prepared in DMSO was then added to the experimental medium in order to reach concentrations from 0.1 to 200 *μ*g/mL. The DMSO concentration of 0.01% was fixed based on the final volume on different wells. For antiestrogenic evaluation, before cell incubation, 17*β*-estradiol 10 nM was added to the wells. Cells treated with DMSO (0.01%) and FBS 10% in RPMI served as solvent and medium controls, respectively. Quercetin (10 nM) was used as the reference/standard. The assay was stopped after 144 h by removing the medium from the wells, fixing the cells with cold trichloroacetic acid 10%, and incubation at 4°C for 1 h. Thereafter, cells were washed four times with tap water and dried. Cells were stained for 30 min with Sulforhodamine-B (SRB) 0.057% (w/v) which was dissolved in 1% acetic acid, rinsed four times with acetic acid 1%, and air-dried. Bound dye was solubilized with Tris base 10 mM (pH 10.5) in a shaker. Finally, aliquots were read in a Biotek EL800 absorbance reader (Winooski, USA) at 510 nm. The results related to estrogenic activity were expressed as mean ± standard error of the mean (SEM) of the proliferative effect (PE), which was calculated according to Schilirò et al. [25]:* PE = max cell number of sample/cell number of DMSO control*. The estrogenic activity of a sample was determined as the relative proliferative effect (RPE%). The RPE compares the maximum proliferation induced by a sample with that induced by 17*β*-estradiol:* RPE% = [PE for sample/PE for 17β-estradiol]* ×* 100* [26].

### 2.4. *In Vivo* Experiments

#### 2.4.1. Animals

Healthy female albinos Wistar rats (10–12 weeks old) weighing ~ 150 g were supplied by the production facility of the Animal Physiology Laboratory, University of Yaounde 1 (Cameroon). All rats were housed in clean plastic cages at the room temperature and lit with natural light. They were given free access to tap water and free-soy rat chow. The composition of animal diet was corn (36.7%), bone flour (14.5%), wheat (36.6%), fish flour (4.8%), crushed palm kernel (7.3%), sodium chloride (0.3%), and vitamin complex (Olivitazol®, 0.01%). All experiments were conducted in accordance with the principles and procedures of the European Union on Animal Care (CEE Council 86/609) guidelines adopted by the Cameroon Institutional National Ethic Committee, Ministry of Scientific Research and Technology Innovation (Reg. number FWA-IRD 0001954).

#### 2.4.2. The 3-Day Uterotrophic Assay

The estrogenic activity of* L. acida* extract was evaluated using a classical 3-day uterotrophic assay in ovariectomized adult rats [27]. Thirty female Wistar rats were ovariectomized (OVX) under ketamine and valium anesthesia (10 mg/kg and 50 mg/kg BW* i.p.*, resp.) and randomly grouped into six groups of five rats each, after 14 days of endogenous hormonal decline. The first group (OVX) received 10 mL/kg distilled water. The second group served as positive control and received 17*β*-estradiol valerate (E2V) at the dose of 1 mg/kg BW/day. The four remaining groups were treated with* L. acida* ethanolic extract at the doses of 50, 100, 200, and 300 mg/kg BW. Animals were orally treated for 3 days; then vaginal smears were analyzed and animals were sacrificed by decapitation under light anesthesia (10 mg/kg BW diazepam and 50 mg/kg BW ketamine* i.p.*). Estrogen target organs (uterus, vagina, and mammary gland) were collected and fixed in 10% formaldehyde for histomorphological analysis. Prior to the fixation, wet uteri were trimmed of fat and weighed. As endpoints, the uterine wet weight, uterine protein and glycogen levels, uterine water content, and uterine and vaginal epithelial heights as well as mammary gland alveolar and ductal structures were assessed.

#### 2.4.3. The Postmenopausal-Rat Model of Osteoporosis

Twenty-five rats were either sham operated (Sham) or bilaterally ovariectomized (OVX) using the dorsal approach. Seven days later, animals were further distributed in five different groups (*n* = 5) and treated by gavage once daily (9:00-10:00 a.m.) for 84 consecutive days as follows: Sham and one OVX group received vehicle (distilled water), while the third group received 1 mg/kg of E2V. The two further groups received* L. acida* extract at the doses of 50 and 200 mg/kg BW. Animals were weighed weekly. Twenty-four hours after the last administration and following a 12 h of overnight fasting, animals were sacrificed under light anesthesia. Blood samples were taken and centrifuged at 3500 rpm (15 min at 4°C) to obtain serum samples which were kept at −15°C for the determination of alkaline phosphatase activity as well as inorganic phosphorus levels. The uterus, vagina, femur, tibia, third lumbar vertebrae (VL-3), mandible (the interradicular septum of the second molar) [28], liver, lungs, kidneys, stomach, adrenal, spleen, and heart were dissected out and cleaned of all soft tissues. Prior to immersion-fixation of organs in the 10% formaldehyde solution for histological analysis, they were weighted. Fresh bone (femur and tibia) volumes were measured using a plethysmometer and their density was calculated using the formula: density = [bone wet weight (kg) × 1000 (kg/mm^3^)/volume of bone (mm^3^)] as described by Lee et al. [29]. After measuring the above parameters, bones were dried at 200°C for 24 h and weighed again. Further, they were calcined and the ashes obtained were dissolved in deionized water (0.5 g per 2 mL) and kept in −15°C for the measurement of calcium and inorganic phosphorus content. The femur, VL-3, and mandible were successively fixed in 10% formalin for 1 week and decalcified in 10% nitric acid [30] for histological analysis.

### 2.5. Cytological and Histological Analysis

Vaginal smears were taken using an eyedropper containing 10 mL of NaCl 0.9%, placed on ringed slides, fixed in ethanol 95% for 30 min, and stained with a Papanicolaou method [31]. Cellular types were observed under a light microscope using 400 magnifications. To determine the histomorphological changes in mammary gland, uterus, vagina, femur, VL-3, mandibular bone, liver, lungs, and kidneys, paraffin-embedded organs were cut to 5 *μ*m tissue sections and stained with hematoxylin and eosin. Stained sections were visualized and images captured using the complete Zeiss equipment consisting of a microscope Axioskop 40 connected to a computer where the images were transferred and analyzed with the MRGrab1.0 and Axio Vision 3.1 software, all provided by Zeiss (Hallbergmoos, Germany).

### 2.6. Biochemical Analysis

Total protein and glycogen levels in uteri were determined using the colorimetric method described by Gonal et al. [32] and Montgomery [33], respectively. The total calcium and inorganic phosphorus levels in bones were determined using reagent kits purchased from fortress Diagnostic (Muckamore, United Kingdom) and Human Gesellschaft (Germany). Serum alkaline phosphatase activity in serum was measured using reagent kits purchased from BIOLABO (Maizy, France).

For hematological analysis, white blood cell count, lymphocytes, monocytes, granulocytes, red blood cells (RBC) count, hematocrit (Ht), hemoglobin (Hb), mean corpuscular volume (MCV), mean corpuscular hemoglobin (MCH), mean corpuscular hemoglobin concentration (MCHC), and platelet count were evaluated using a Humacount 30^TS^ Automated Hematology Analyzer from Human Diagnostics Worldwide (Wiesbaden, Germany).

### 2.7. Statistical Analysis

Results were presented as means ± standard error of mean (SEM).* In vitro* experiments were performed in triplicate and repeated three times. All formulas and function were calculated with Microsoft Excel software. Data analysis was performed with GraphPad Prism 5.0 Software, using the ANOVA test followed by Dunnett's post hoc test. Differences were considered significant at a probability level of 5% (*p* < 0.05).

## 3. Results

### 3.1. Results of Phytochemical Analyses

Preliminary phytochemical analysis showed that the ethanolic extract of stem bark of* L. acida* ethanolic possess phenols, flavonoids, saponins, tannins, and alkaloids. Quantitative phytochemical analysis of this extract revealed the quantity per g of dried extract of total phenols (786.75 ± 82.33), flavonoids (250.61 ± 48.17), and alkaloids (31.64 ± 5.63) (Table 1).

### 3.2. *In Vitro* Estrogenicity Assessment

#### 3.2.1. Cytotoxicity

**Figure 1:**
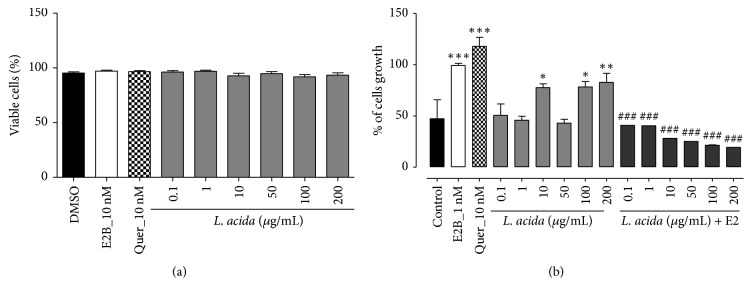
Effects of* L. acida* ethanol extract on MCF-7 cells proliferation viability (a) and growth (b). Its effect was investigated by measuring E-screen assay. The relative MCF-7 cells yields (PE) were measured in the presence of DMSO (0.01%), 17*β*-estradiol benzoate (E2B, 10 nM), quercertin (10 nM), and* L. acida* extract (from 0.1 to 200 *μ*g/mL). PE = max cell number of sample/cell number of DMSO control; ^*∗*^*p* < 0.05, ^*∗∗*^*p* < 0.01 and ^*∗∗∗*^*p* < 0.001 as compared to the DMSO control. ^###^*p* < 0.001 as compared to the E2B control.

Ethanolic extract of* Lannea acida* did not induce cytotoxic effects in MCF-7 cells at tested concentrations (Figure 1(a)).

#### 3.2.2. E-Screen Assay

Effects of* L. acida* ethanolic extract on MCF-7 cells proliferation are depicted in Figure 1(b). It can be observed that 17-*β* estradiol benzoate (1 nM) and quercetin (10 nM) induced a significant (*p* < 0.001) increase of MCF-7 cells yield. The* L. acida* ethanol extract induced a significant increase of MCF-7 cells yield at concentrations of 10 (*p* < 0.05), 100 (*p* < 0.05), and 200 (*p* < 0.01) *μ*g/mL as compared to DMSO control. Further, a significant (*p* < 0.001) and concentration-dependent antiestrogenic effect was noted with* L. acida *extract when administered with E2B.

### 3.3. Results of the 3-Day Treatment with* L. acida*

#### 3.3.1. Effects on the Uterine Wet Weight and Uterine Content Parameters

As shown in Table 2, the 3-day oral administration of* L. acida* extract induced a significant increase in uterine wet weight and uterine water content at all tested doses as compared to OVX group. Similarly, the uterine total protein and glycogen levels significantly increased at all the tested doses except at the dose of 300 mg/kg. The maximum increase was observed at the dose of 200 mg/kg BW; however, this increase remained much lower than in E2V-treated group.

#### 3.3.2. Effects on the Uterine Epithelium

**Figure 2:**
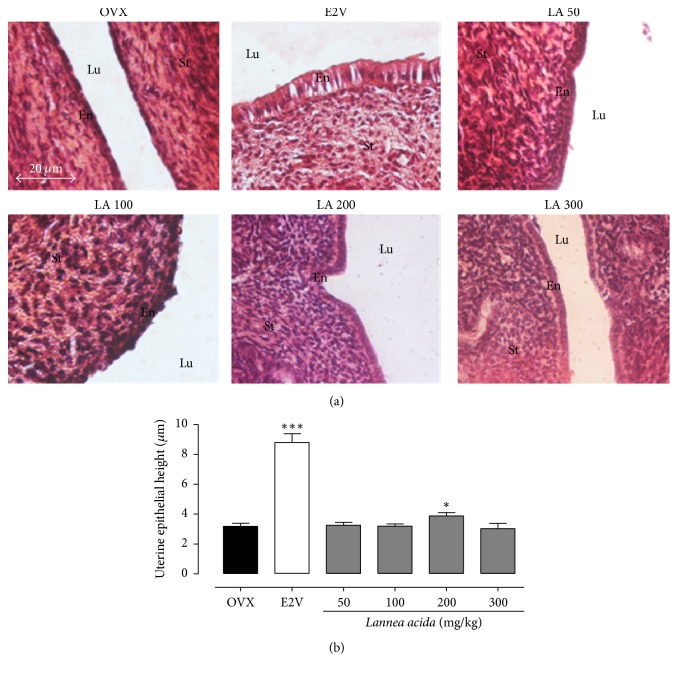
Effects of a 3-day treatment with* L. acida* ethanol extract on microphotographs (hematoxylin-eosin staining, 400x) (a) and graphical representation of uterine epithelial height (b). OVX = OVX animals treated with distilled water as vehicle; E2V = OVX animals treated with 17*β*-estradiol valerate at 1 mg/kg BW;* Lannea acida* = OVX animals treated with* L. acida* ethanol extract at doses of 50, 100, 200, and 300 mg/kg BW. ^*∗*^*p* < 0.05 and ^*∗∗∗*^*p* < 0.001 as compared with OVX control. Lu: uterine lumen; En: endometrium; St: stroma.

The 3-day treatment with the ethanolic extract of* L. acida* induced a significant (*p* < 0.05) increase in uterine epithelial height only at dose of 200 mg/kg BW (from 3.17 ± 0.21 to 3.87 ± 0.22 *μ*m). However this increase remained much lower than that induced by E2V at the dose of 1 mg/kg BW, which showed a 3-fold (*p* < 0.001) increase of uterine epithelial height (Figure 2(b)). The photomicrographs of uteri of OVX animals showed a low cuboidal epithelium, while in the E2V treated group, all structures are hypertrophic; the endometrium is lined by tall columnar cells with squamous metaplasia and atypic mitotic pattern surrounded by anaplastic epithelial nuclei (Figure 2(a)). Microphotographs of uteri of animals receiving* L. acida* ethanol extract at the dose of 200 mg/kg displayed an endometrium consisting of tall cuboidal single-layered epithelial cells with abundant mitotic figures and necrosis; however, this effect is less than those observed in E2V-treated group.

#### 3.3.3. Effects on Vagina

**Figure 3:**
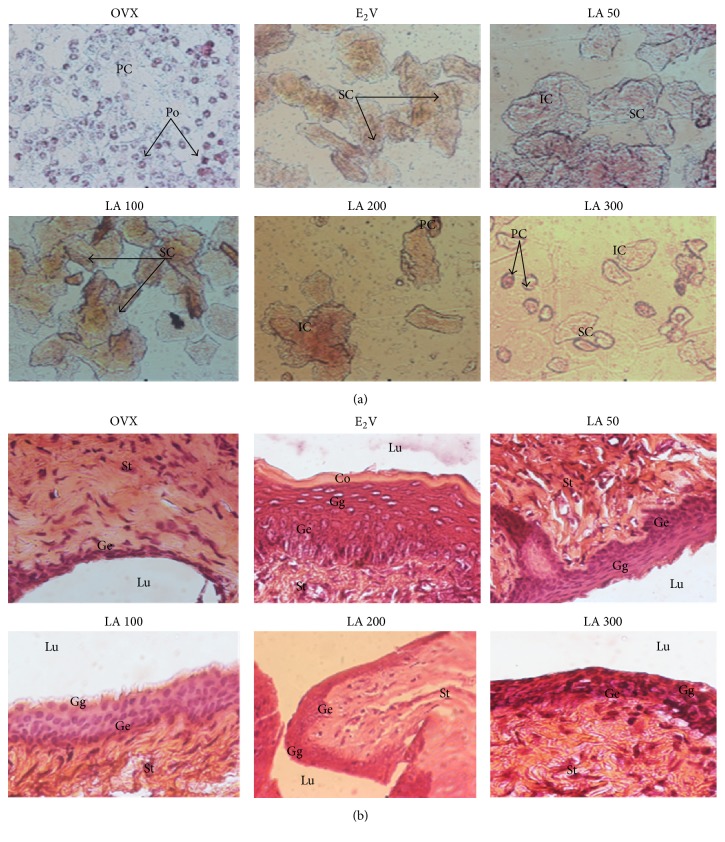
Effects of a 3-day treatment with* L. acida* ethanol extract on the vaginal epithelium: smear (Papanicolaou staining, 400x) (a) and microphotographs (hematoxylin-eosin staining, 400x). OVX = OVX animals treated with distilled water as the vehicle; E2V = OVX animals treated with 17*β*-estradiol valerate at 1 mg/kg BW;* Lannea acida* = OVX animals treated with* L. acida* ethanol extract at doses of 50, 100, 200, and 300 mg/kg BW. Po = polynuclear cells, PC = parabasal cells, IC = intermediate cells, SP = superficial cells, Lv = vaginal lumen, Co = stratum corneum, Gg = stratum granulosum, Ge = stratum germinativum, and St: stroma.

As depicted in Figure 3(a) vaginal smears of controls (OVX) had predominantly parabasal and polynuclear cells, corresponding to diestrus. On the other hand, smears of animals treated with E2V and* L. acida* extract at all tested doses suggest that they are in estrus, evidenced by the presence of superficial cells (Figure 3(a)). Furthermore, the microphotographs of vaginal epithelium of the control group (OVX) consist simply of the stratum germinativum (Ge) at the lowest level of approximately the thickness of three to seven cells. After the treatment with E2V (1 mg/kg), the vaginal epithelium became stratified characterized by the presence of stratum germinativum, stratum granulosum (Gg), and stratum corneum (Co) (Figure 3(b)). Following the* L. acida* extract treatment, the vaginal epithelium became hypertrophic and hyperplasic; however compared to E2V there are fewer cell layers and it failed to induce a cornification.

**Figure 4:**
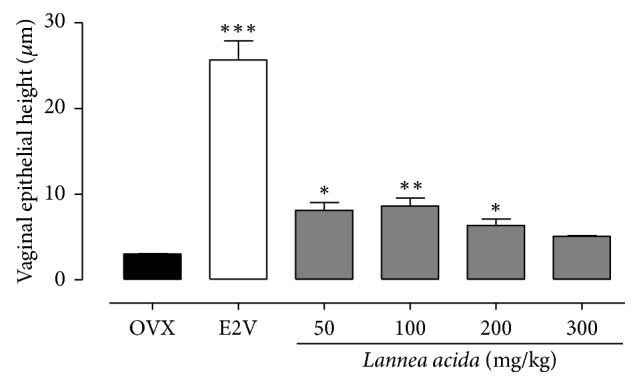
Effects of a 3-day treatment with* L. acida* ethanol extract on the vaginal epithelial height. OVX = OVX animals treated with distilled water as the vehicle; E2V = OVX animals treated with 17*β*-estradiol valerate at 1 mg/kg BW;* Lannea acida* = OVX animals treated with* L. acida* ethanol extract at doses of 50, 100, 200, and 300 mg/kg BW. ^*∗*^*p* < 0.05, ^*∗∗*^*p* < 0.01, and ^*∗∗∗*^*p* < 0.001 as compared with OVX control.

The graphical representation of the vaginal epithelial height (Figure 4) shows that E2V induced a 5-fold (*p* < 0.001) increase of vaginal epithelial height.* L. acida* ethanolic extract significantly (*p* < 0.01) and in a bell shaped dose response manner increased vaginal epithelial height. The maximum 2.8-fold increment was obtained with the dose of 100 mg/kg BW (from 3.03 ± 0.04 to 8.6 ± 0.92 *μ*m) as compared to the OVX group.

#### 3.3.4. Effects on Mammary Glands

**Figure 5:**
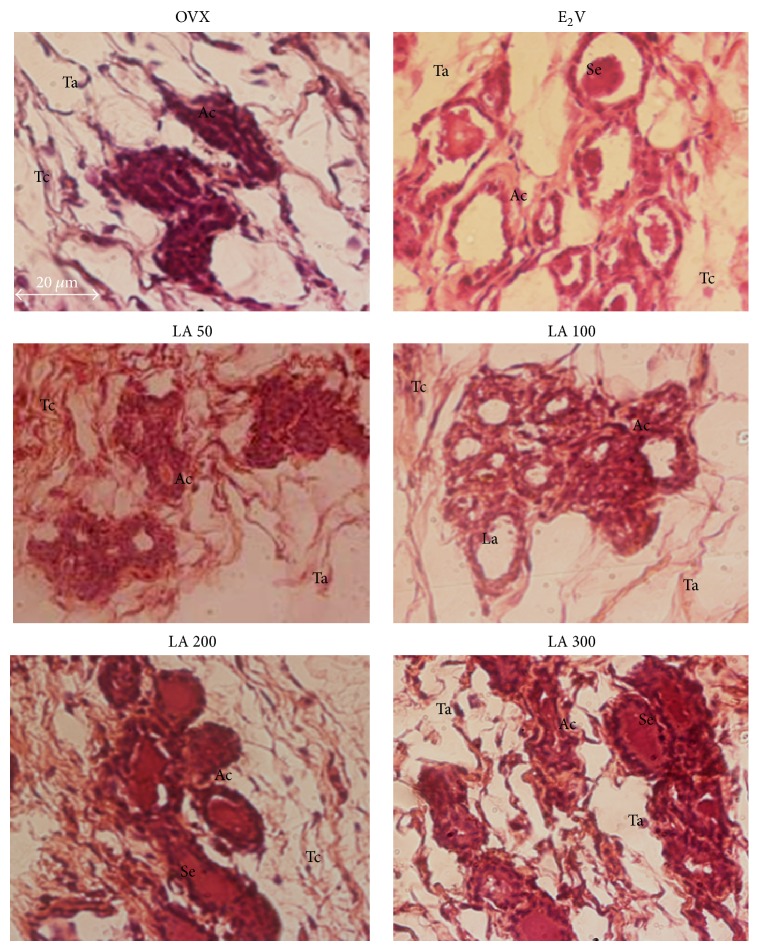
Effects of a 3-day treatment with* L. acida* ethanol extract on mammary glands: microphotographs of hematoxylin-eosin staining (400x). OVX = OVX animals treated with distilled water as the vehicle; E2V = OVX animals treated with 17*β*-estradiol valerate at 1 mg/kg BW; LA = OVX animals treated with* L. acida* ethanol extract at doses of 50, 100, 200, and 300 mg/kg BW. La = lumen of alveoli; Ep = alveoli epithelium; At = adipose tissue; Se = eosinophil secretion.

Figure 5 presents sections of mammary glands. Ovariectomy induced an atrophy of mammary glands materialized in OVX-histological sections by a modest alveolar development, a small cluster of densely packed epithelial cells without luminal formation, the loss of the gland parenchyma (Tc), and the ductular and alveolar components, while adipocyte tissue (At) appears prominent. Mammary glands of E2V-treated group present well-formed acinar and luminal structures, an increase in proliferative activity compared to OVX group such as increase of the diameter and the lumen of alveoli, abundant eosinophil secretion (Se) in lumen of alveoli, and further compression of stromal fat. Similar changes were observed following a 3-day treatment with* L. acida* extract at all tested doses but only doses of 200 and 300 mg/kg BW presented an eosinophil secretion in their lumens.

### 3.4. Results of the 84-Day Treatment with* L. acida*

#### 3.4.1. Effects on Estrogen Target Organs

As depicted in Table 3, bilaterally oophorectomy significantly (*p* < 0.001) decreased the uterine wet weight (610.13%) and uterine (388.57%) and vaginal (419.4%) epithelial height as compared to the Sham operated group. Following E2V treatment, the uterine wet weight (694.65%) and the uterine (397.39%) and vaginal (419.4%) epithelial height increased as compared to OVX group.* L. acida* extract induced a slight but significant (*p* < 0.05) increase of uterine epithelial height at the dose of 50 mg/kg BW (30.82%) compared to OVX control.

#### 3.4.2. Effects on Bone Weight and Density

**Figure 6:**
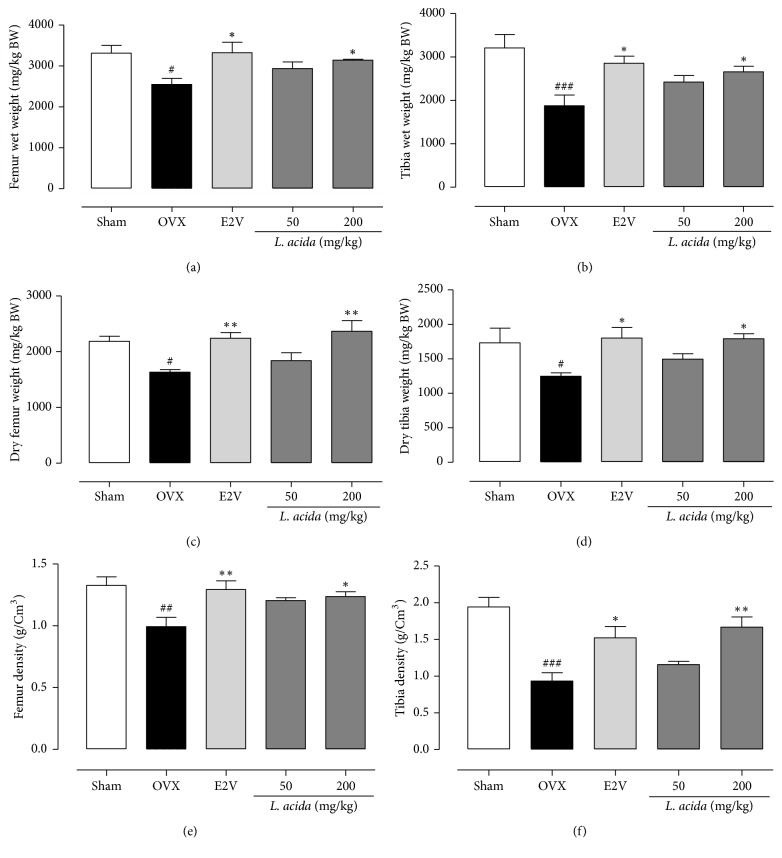
Protective effects of* L. acida *ethanol extract on bone loss: in both femur and tibia wet weight ((a) and (b)), dried weight ((c) and (d)), and density ((e) and (f)). Sham (sham operated) and OVX (ovariectomized) animals received the vehicle (distilled water). E2V = OVX animals treated with 1 mg/kg of 17*β*-estradiol valerate;* Lannea acida* = OVX animals treated with ethanol extract of* Lannea acida* at doses of 50 and 200 mg/kg BW. Data are expressed as mean ± SEM (*n* = 5). ^*∗*^*p* < 0.05 and ^*∗∗*^*p* < 0.01 compared with control; ^#^*p* < 0.05, ^##^*p* < 0.01, and ^###^*p* < 0.001 compared with Sham.

As shown in Figure 6 ovariectomy induced a significant decrease in femur (*p* < 0.05) and tibia (*p* < 0.001) wet weight (Figures 6(a) and 6(b)) and a significant (*p* < 0.05) decrease of dried femur and tibia weight as well as the femur (33.78%, *p* < 0.01) and tibia (38.83%, *p* < 0.001) densities as compared to sham operated rats. E2V and* L. acida* extract at the dose of 200 mg/kg induced a significant (*p* < 0.05) increase of femur and tibia wet and dried weights as compared to OVX group. Furthermore, significant (*p* < 0.05) increases of femur and tibia densities were also observed following administration of E2V and* L. acida* extract at the dose of 200 mg/kg as compared to OVX (Figures 6(e) and 6(f)).

#### 3.4.3. Effects on Body and Organ Weights

After 84 days of treatment period, a significant (*p* < 0.05) increase of body weight was observed in OVX and* L. acida* extract (50 mg/kg) groups (Table 4). The E2V and* L. acida* extract at the dose of 200 mg/kg treatments prevented this body weight increase.

The different treatments did not induce change in the organ wet weights, except the relative weights of lungs and kidneys that increased following the 84-day treatment with* L. acida* extract at the dose of 200 mg/kg.

#### 3.4.4. Biochemical Bone Parameters

Table 5 shows the effects of* L. acida* extract or E2V treatment on serum and bone biochemical parameters. Ovariectomized rats showed a significant increase (42.29%; *p* < 0.001) in the serum alkaline phosphatase (ALP) activity as compared to Sham rats. E2V treatment significantly decreased (21.29%, *p* < 0.05) the ALP activity as compared to the OVX controls. The* L. acida* ethanol extract induced a similar effect by decreasing ALP activity at all tested doses, although being only significant at the dose of 50 mg/kg BW/day (31.49%, *p* < 0.001).

Concerning calcium levels in tibia and femur, no significant changes were observed between OVX, Sham, and E2V groups. However, a significant (*p* < 0.05) increase in the calcium level was observed in rats treated with* L. acida* 200 mg/kg as compared to OVX control.

Regarding inorganic phosphorus (IP) levels in tibia and femur ashes, a decrease of IP in tibia (*p* < 0.001) and femur (nonsignificant) was observed in OVX rats as compared to sham operated controls. E2V treatment significantly (73.03%, *p* < 0.05) increased IP in femur compared to OVX controls. Treatment with* L. acida* extract increased (*p* < 0.05) IP levels in femur (50.86%) and in tibia (61.27%) ashes at the dose of 200 mg/kg.

#### 3.4.5. Effects on the Hematological Parameters

Lymphocytes, red blood cell count, and hemoglobin level as well as hematocrit increased in ovariectomized rats as compared to sham operated rats after 84 days of experimentation although these values remain in the normal ranges (Table 6). The E2V-treatment prevented this blood cell increment, although being significant only in the hematocrit parameter.* L. acida* extract also protected rat against the ovariectomy-induced increase in blood cells, evidenced by a significant reduction of lymphocytes, granulocyte, hemoglobin level, and hematocrit.

#### 3.4.6. Effects on the Microarchitecture of Some Organs

**Figure 7:**
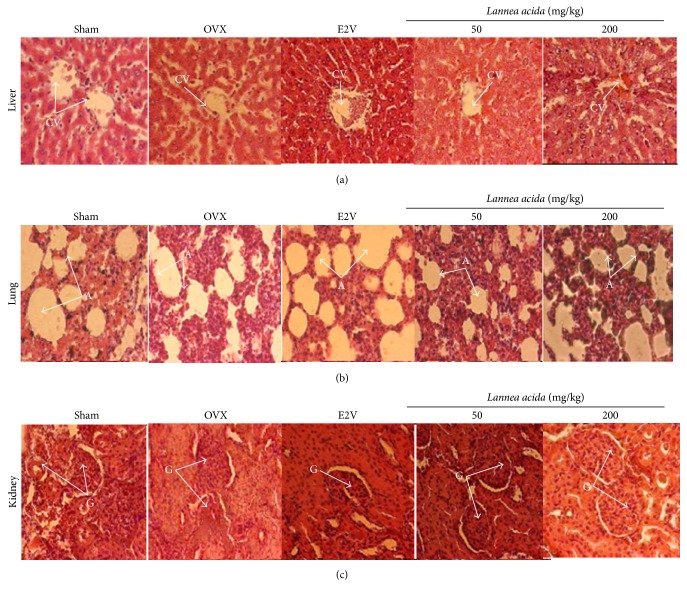
Effects of 11 weeks of treatment with ethanol extract of* Lannea acida* (LA) or E2V on liver (a), lung (b), and kidney (c) of sham operated or ovariectomized rats. Sham (sham operated) and OVX (ovariectomized) animals received the vehicle (distilled water). E2V = OVX animals treated with 1 mg/kg of 17*β*-estradiol valerate;* Lannea acida* = OVX animals treated with ethanol extract of* Lannea acida* at doses of 50 and 200 mg/kg BW. Data are expressed as mean ± SEM (*n* = 5). CV = central vein, A = alveoli; G = glomeruli.

**Figure 8:**
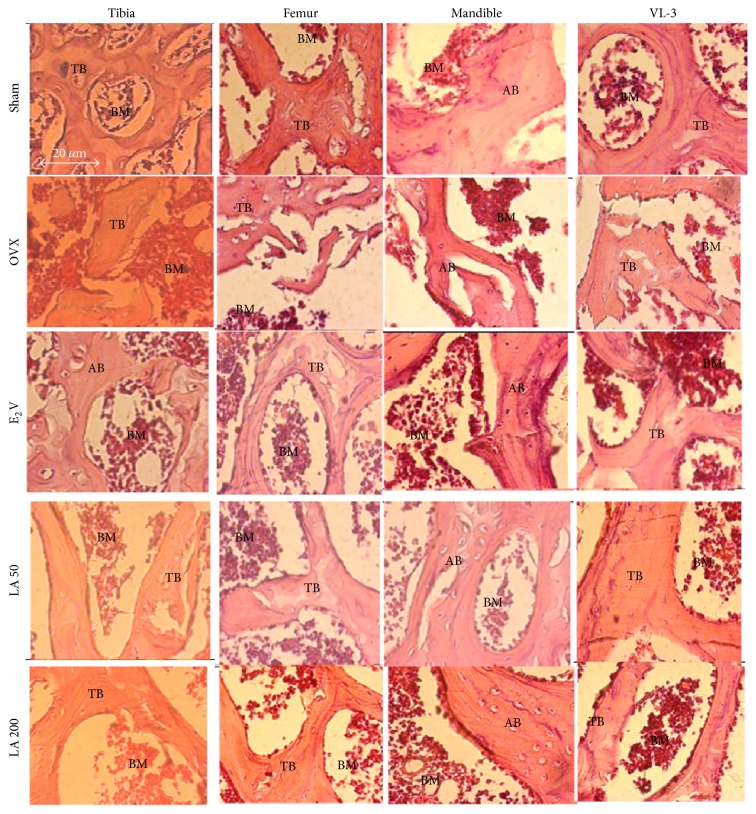
Protective effects of* L. acida* ethanol extract on histomorphologic (100x magnification) of tibia, femur, VL-3, and mandible. Sham (sham operated) and OVX (ovariectomized) animals received the vehicle (distilled water). E2V = OVX animals treated with 1 mg/kg of estradiol valerate;* Lannea acida* = OVX animals treated with ethanol extract of* Lannea acida* at doses of 50 and 200 mg/kg BW. Data are expressed as mean ± SEM (*n* = 5). TB = trabecular bone; AB = alveolar bone; and BM = marrow bone.

No alterations in the microarchitecture of liver, lungs, and kidneys were noted in this work (Figure 7). However, the femur, tibia, VL-3, and mandible microarchitectures of OVX rats showed bone marrow disparities into the trabecular network (Figure 8). E2V and* L. acida* ethanol extract (200 mg/kg) treatments prevented bone resorption, evidenced by the inhibition of bone marrow loss into the trabecular network.

## 4. Discussion

The present work aimed to investigate the estrogenic and bone loss preventive effects of the ethanol extract of* Lannea acida*, a plant used in the Cameroonian traditional system to treat many ailments including gynaecological problems and rheumatism. To evaluate the* in vitro* estrogenic effect of* L. acida*, a suitable biological screening described by Soto et al. [34] and reported in our previous study [24] has been used. The basic principle of this assay is to compare the MCF-7 cells yield following treatment with tested substances with those obtained after estradiol treatment. In this study, the* L. acida* ethanol extract induced a significant increase of MCF-7 cells proliferation at concentrations of 10, 100, and 200 *μ*g/mL as compared to DMSO control. The MCF-7 cells proliferation is known as a hallmark of estrogenicity [34], suggesting that* L. acida* have phytoconstituents that mimic estrogenic effects. Indeed, Resende et al. [26] reported that a relative proliferative effect (RPE) ≥ 80% corresponds to a possible agonistic activity to ER*α*.* L. acida* induced a RPE > 80% at the concentration of 100 and 200 *μ*g/mL. Consequently, flavonols detected in* L. acida* ethanol extract might bind ERs and induced cell proliferation. However, this effect could also be an ER-independent effect, due to the fact that MCF-7 cells express ERs, aromatase, and 5 *α*-reductase enzymes, which permit it to elicit estrogenic response involving both genomic and nongenomic pathways [25]. Although an ER binding assay was not assessed in this study, we can hypothesize that flavonoids reported in* L. acida* could be responsible for the MCF-7 cells proliferation and are potential phytoestrogens. In fact, 4 flavonoids, named as 6,7-(2′′,2′′-dimethyl chromeno)-8-*γ*,*γ*-dimethyl allyl flavanone, 3′,4′-dihydroxy-7,8 (2′′,2′′- dimethyl chromeno)-6-*γ*,*γ*-dimethyl allyl flavonol, 7-methyltectorigenin, and irisolidone, have been isolated from barks of the acetone extract of* Lannea acida* [18]. Irisolidone and tectorigenin (an analogue of 7-methyltectorigenin resulting from the hydrolyzing by intestinal bacteria) have been reported to exhibit estrogenic effects in E-screen assay, as they have induced MCF-7 cells proliferation at concentrations of 0.1, 1, and 10 *μ*M [35]. Therefore they could at least partly account for the proliferative activity of* L. acida* on MCF-7 cells observed in this study. Moreover, the concomitant administration of* L. acida* extract with estradiol leads to the decrease of the MCF-7 cells proliferation, suggesting that some of the phytoconstituents of* L. acida* entered in competition with E2V for ERs. The phytoestrogens are well known to bind ERs with ~ 1000 times lower affinity than estradiol [36].

Numerous studies showed that estrogen deficiency is accompanied with a marked atrophy of estrogen target organs such as uterus, vagina, and mammary glands [23, 24, 37, 38]. The same observation was done in this study; ovariectomy significantly reduced vaginal epithelium to one cell layer: the stratum germinativum. The uterine wet weight as well as uterine and vaginal epithelial heights decreased dramatically after ovariectomy. As expected, estradiol induced a significant increase in the uterine wet weight and in uterine and vaginal epithelial heights after a 12-week treatment. The uterotrophic effects of estrogen have been attributed to water imbibition or endometrial cells proliferation [39, 40]. These effects have been reported to be mediated via ER*α* as demonstrated by the lack of uterine stimulation and mitotic growth responses in *α*ERKO mice [41].* Lannea acida* ethanol extract induced an estrogen-like effect, evidenced by a significant increase in the uterine wet weight and uterine and vaginal epithelial heights at all tested doses (except with the dose of 300 mg/kg in some parameters) in the 3-day uterotrophic test. These results are in line with the* in vitro* results obtained in this study and strengthen our hypothesis that the flavonoids detected in* L. acida* could bind ERs and trigger the genomic mechanism that produces estrogen-like effects. Interestingly, the 12-week treatment of ovariectomized rats with* L. acida* extract did not exhibit estrogenic effect, a part of a slight increase of the uterine epithelial height observed with the dose of 50 mg/kg, indicating that the estrogen-like effects of* L. acida* extract are time-dependent. These types of effects are specific to phytoestrogens. Indeed, flavonoids are well known phytoestrogens with mixed effects [23, 24, 38, 42].

The postmenopausal-rat model of osteoporosis has been used for decade to characterize natural substances. It shares many characteristics of postmenopausal women bone loss and therefore it is a suitable model for postmenopausal osteoporosis [43–45]. Following the 12 weeks of administration period, the body weight of ovariectomized rats significantly increased as compared to other groups. This result corroborates previous observations [45, 46] and could be explained by an increase in adipose deposition. E2V (1 mg/kg) and* L. acida* extract at the optimal dose of 200 mg/kg prevented this body weight gain, probably by mechanisms involving ERs [47]. In fact, estrogen is known to reverse ovariectomy-induced body weight gain. Furthermore, ovariectomy induced a significant decrease of tibia and femur (weight and dried) weights as well as tibia and femur densities. Moreover, a significant decrease in inorganic phosphate level in tibia and a significant increase of serum alkaline phosphatase activity were noted. All these events suggest that ovariectomy enhanced the rate of bone turnover and the experimental osteoporosis was set up in rats. Indeed, the alkaline phosphatase activity is a biomarker of osteoblastic activity associated with bone formation [48]. It is found to increase in osteoporosis and other bone metabolic disorders [41]. Treatment with E2V and* L. acida* extract at the dose of 200 mg/kg increased bone (tibia and femur) wet and dried weights as well as bone densities. In addition, these treatments decreased the serum alkaline phosphatase activity and increase the bone inorganic phosphorus content. The aforesaid results suggest that* L. acida* extract reduced bone turnover. The potential preventive effects of estrogen and estrogen-like substances on bone loss have been attributed to their ability to bind ERs on the osteoclast cells and to provoke release of chemical mediators and reduction of the osteoclastic activity [49].

Bone histomorphometry is a technique often used to provide information about bone weight gain or loss in untreated and treated diseases [28, 45, 46, 50]. These studies have showed that ovariectomy resulted in increased bone turnover with resorption exceeding formation. This imbalance is known to lead to progressive loss in bone mass and eventually osteoporosis [51]. In accordance with this, our results showed that ovariectomy induced microarchitectural alterations of all the studied bones (VL-3, mandible, femur, and tibia). However, treatments with E2V and* Lannea acida* extract improved the bone tissue microarchitecture, restoring both cortical and compact bone structure. The same results were observed by Njamen et al. [52] and Zingue et al. [45]. The flavonoids/isoflavonoids of* Lannea acida* could account for this beneficial effect on bone since phytoestrogens have been reported to play an important role in the bone formation through the activation of the ER*β* localized on osteoblast cells [53].

## 5. Conclusion

The ethanol extract of* Lannea acida* possesses* in vitro* and* in vivo* estrogenic effects, materialized by the proliferation of MCF-7 cells and the increase in uterine wet weight and uterine and vaginal epithelial heights. Results also suggest that long-term (3 months) treatment with* L. acida* extract could prevent estrogen decline-related bone mass loss, microarchitecture alterations, and demineralization. The dose of 200 mg/kg BW/day was the optimal dose. Taken altogether, this finding provides the evidence that* L. acida* is a potential alternative for the prevention of postmenopausal osteoporosis which occurs as a consequence of estrogen decline at menopause. In-depth phytochemical investigations are needed to isolate the active principles of* L. acida* and understand the precise mechanism by which it induced estrogenic effect.

## Figures and Tables

**Table 1:** Quantitative analyses of selected phytochemicals present in *L. acida *ethanolextract.

Number	Phytochemical class	Concentration in *L. acida* extract
1	Total phenols	786.75 ± 82.33 mg eq quercetin
2	Flavonoids	250.61 ± 48.17 mg eq quercetin
3	Alkaloids	31.64 ± 5.63 mg eq berberine

Total phenols and flavonoids are expressed in mg eq quercetin/g of dried extract while alkaloids content is expressed in mg eq berberine/g of dried extract. Data are represented as mean ± SEM of triplicates from at least three independent experiments.

**Table 2:** Effects of the ethanol extract of *Lannea acida* on uterus.

Groups	Uterine wet weight (mg/kg)	Water content in uterus WW/DW	Total protein levels in uterus (mg/mL)	Glycogen level in uterus (mg/mL)
OVX	298.75 ± 33.55	0.13 ± 0.014	0.1087 ± 0.0002	5.43 ± 0.11
E2V	2022.63 ± 91.81^*∗∗∗*^	0.41 ± 0.058^*∗∗∗*^	0.1114 ± 0.0003^*∗∗*^	9.21 ± 0.21^*∗∗*^
*Lannea acida* (mg/kg)				
50	507.64 ± 10.42^*∗∗*^	0.20 ± 0.008^*∗*^	0.1096 ± 0.0003^*∗*^	6.71 ± 0.42^*∗*^
100	424.73 ± 37.48^*∗*^	0.31 ± 0.025^*∗∗*^	0.1095 ± 0.0002^*∗*^	7.3 ± 0.25^*∗*^
200	553.38 ± 43.72^*∗∗*^	0.29 ± 0.036^*∗∗*^	0.1105 ± 0.0001^*∗∗*^	6.9 ± 0.53^*∗*^
300	424.69 ± 31.48^*∗*^	0.23 ± 0.032^*∗*^	0.1090 ± 0.0002	6.11 ± 0.3

OVX = ovariectomized animals that received the vehicle (distilled water); E2V = OVX animals treated with 1 mg/kg of 17*β*-estradiol valerate; *Lannea acida* = OVX animals treated with ethanol extract of *Lannea acida* at doses of 50, 100, 200, and 300 mg/kg BW. WW = wet weight; DW = dried weight. Data are expressed as mean ± SEM (*n* = 5); ^*∗*^*p* < 0.05, ^*∗∗*^*p* < 0.01, and ^*∗∗∗*^*p* < 0.001 as compared to the OVX group.

**Table 3:** Effects of the ethanol extract of *Lannea acida* on estrogen target organs in postmenopausal-rat model after 12 weeks of treatment.

Items	Sham	Ovariectomized rats			
		OVX	E2V	*Lannea acida* (mg/kg BW)	
				50	200
Uterine wet weight (mg/kg)	1761.34 ± 174.20	248.03 ± 19.56^###^	1970.98 ± 203.67^*∗∗*^	226.36 ± 16.22	277.37 ± 17.63
Uterine epithelial height (*µ*m)	9.88 ± 0.29	2.02 ± 0.06^###^	10.05 ± 1.29^*∗∗*^	5.79 ± 1.75^**∗**^	2.79 ± 0.19
Vaginal epithelial height (*µ*m)	21.83 ± 1.82	4.20 ± 0.29^###^	46.01 ± 0.85^*∗∗*^	5.98 ± 0.29	6.38 ± 0.35

Sham (sham operated) and OVX (ovariectomized) animals received the vehicle (distilled water); E2V = OVX animals treated with 1 mg/kg of 17*β*-estradiol valerate; *Lannea acida* = OVX animals treated with ethanol extract of *Lannea acida *at doses of 50 and 200 mg/kg BW. Data are expressed as mean ± SEM (*n* = 5); ^*∗*^*p* < 0.05 and ^*∗∗*^*p* < 0.01 as compared to the OVX group. ^###^*p* < 0.001 as compared to the Sham group.

**Table 4:** Effects of *L. acida* ethanol extract on body weight and relative organ weights after 12 weeks of treatment in postmenopausal-rat model.

Items	Sham	Ovariectomized rats			
		OVX	E2V	*Lannea acida* (mg/kg BW)	
				50	200
*Body weight (g)*					
Initial	155.80 ± 5.67	145.80 ± 2.75	147.00 ± 4.49	153.60 ± 4.43	146.10 ± 5.23
Final	182.10 ± 8.51	203.10 ± 3.22^#^	185.17 ± 5.13	199.74 ± 6.17^#^	183.14 ± 7.79
*Organ weights (mg/kg)*					
Liver	32283.86 ± 845.01	30918.79 ± 701.81	31590.70 ± 948.29	31055.44 ± 905.08	28137.84 ± 1924.21
Lungs	7648.59 ± 166.14	8525.59 ± 243.24	7834.56 ± 374.67	8799.39 ± 472.94	11380.12 ± 1053.63^*∗∗*^
Kidneys	5013.12 ± 107.64	5127.95 ± 59.69	5366.69 ± 284.57	5105.20 ± 171.53	5989.34 ± 275.03^*∗*^
Stomach	10569.99 ± 277.86	11892.14 ± 761.88	12379.29 ± 848.28	12554.57 ± 1102.40	13042.20 ± 1229.32
Spleen	3442.94 ± 82.54	3196.14 ± 67.20	3526.94 ± 324.13	3626.61 ± 135.77	3618.77 ± 166.85
Adrenals	392.17 ± 147.31	211.02 ± 27.70	221.09 ± 24.89	213.17 ± 5.20	197.70 ± 11.40
Heart	3119.72 ± 24.16	3432.90 ± 120.48	3194.82 ± 151.18	3475.69 ± 166.82	3527.98 ± 36.96

Sham (sham operated) and OVX (ovariectomized) animals received the vehicle (distilled water); E2V = OVX animals treated with 1 mg/kg of 17*β*-estradiol valerate; *Lannea acida* = OVX animals treated with ethanol extract of *Lannea acida *at doses of 50 and 200 mg/kg BW. Data are expressed as mean ± SEM (*n* = 5); ^*∗*^*p* < 0.05 and ^*∗∗*^*p* < 0.01 as compared to the OVX group. ^#^*p* < 0.01, initial body weight compared to the final one.

**Table 5:** Effects of *Lannea acida* ethanol extract after 12 weeks of oral treatment in some biomarkers of osteoporosis.

Items	Sham	Ovariectomized rats			
		OVX	E2V	*Lannea acida* (mg/kg BW)	
				50	200
*Alkaline phosphatase (UI/L)*	5.03 ± 0.456	8.72 ± 0.618^*∗∗∗*^	6.86 ± 0.377^*∗*^	5.63 ± 0.096^*∗∗∗*^	7.56 ± 0.330
*Calcium (mmol/L)*					
Tibia	0.045 ± 0.007	0.028 ± 0.003	0.050 ± 0.011	0.038 ± 0.004	0.028 ± 0.005
Femur	0.03 ± 0.004	0.028 ± 0.002	0.025 ± 0.006	0.032 ± 0.002	0.043 ± 0.005^*∗*^
*Inorganic phosphorus (mmol/L)*					
Tibia	7.24 ± 0.622	2.88 ± 0.122^*∗∗∗*^	3.99 ± 0.347	3.23 ± 0.756	4.64 ± 0.344^*∗*^
Femur	3.99 ± 0.540	2.85 ± 0.384	4.93 ± 0.697^*∗*^	2.72 ± 0.182	4.30 ± 0.482^*∗*^

Sham (sham operated) and OVX (ovariectomized) animals received the vehicle (distilled water); E2V = OVX animals treated with 1 mg/kg of estradiol valerate; *Lannea acida* = OVX animals treated with ethanol extract of *Lannea acida* at doses of 50 and 200 mg/kg BW. Data are expressed as mean ± SEM (*n* = 5); ^*∗*^*p* < 0.05 and ^*∗∗∗*^*p* < 0.001 as compared to the OVX group.

**Table 6:** Effects of *Lannea acida* ethanol extract on hematological parameters after 11 weeks of treatment in postmenopausal-rat model.

Items	Normal ranges	Sham	Ovariectomized rats			
			OVX	E2V	*Lannea acida* (mg/kg BW)	
					50	200
WBC (10^3^/*μ*L)	5–16	4.02 ± 0.42	4.00 ± 0.21	5.62 ± 0.80	2.48 ± 0.14	5.58 ± 0.54
Lymphocytes (%)	65–85	74.42 ± 1.77	83.06 ± 0.46^*∗∗*^	82.6 ± 1.59	85.46 ± 1.148	73.8 ± 2.46^*∗∗*^
Monocytes (%)	0–20	13.52 ± 2.14	10.1 ± 0.33	10.86 ± 0.98	9.05 ± 0.74	11.82 ± 0.99
Granulocytes (%)	0–27	10.15 ± 1.58	7.76 ± 0.83	10.32 ± 1.42	5.5 ± 0.55	13.7 ± 1.69^*∗*^
RBC (10^6^/*μ*L)	5–10	3.65 ± 0.83	7.41 ± 0.18^*∗∗*^	4.97 ± 0.85	4.99 ± 0.99	6.24 ± 0.28
Hematocrit (%)	32–53	25.56 ± 2.99	41.72 ± 1.09^*∗∗*^	31.93 ± 3.02^*∗∗*^	31.60 ± 1.92^*∗∗*^	33.48 ± 1.30^*∗*^
Hemoglobin (g/dL)	12–18	10.48 ± 0.57	14.6 ± 0.54^*∗*^	13.32 ± 0.09	9.56 ± 1.70^*∗∗*^	11.40 ± 0.44
MCV (fL)	52–60	67.80 ± 5.94	56.80 ± 0.58	70.20 ± 7.27	54.2 ± 0.66	68.20 ± 11.84
MCH (pg)	17–29	36.38 ± 8.40	19.62 ± 0.38	32.80 ± 7.35	20.20 ± 1.03	39.92 ± 16.96
MCHC (g/dL)	32–45	50.45 ± 7.21	34.90 ± 0.58	44.00 ± 4.94	37.20 ± 1.60	48.68 ± 11.32
Platelets (10^3^/*μ*L)	200–1100	343.00 ± 42.97	338.8 ± 26.36	485.00 ± 53.95	241.20 ± 45.33	483.00 ± 17.68

Sham (sham operated) and OVX (ovariectomized) animals received the vehicle (distilled water); E2V = OVX animals treated with 1 mg/kg of 17*β*-estradiol valerate; *Lannea acida* = OVX animals treated with ethanol extract of *Lannea acida* at doses of 50 and 200 mg/kg BW. Data are expressed as mean ± SEM (*n* = 5); ^*∗*^*p* < 0.05 and ^*∗∗*^*p* < 0.01 as compared to the OVX group.

## Abbreviations

E2V: Estradiol valerate
ER: Estrogen receptor
ER*α*: Estrogen receptor alpha
ER*β*: Estrogen receptor beta
EtOH: Ethanol
FBS: Fetal bovine serum
HEPES: 4-(2-Hydroxyethyl)-1-piperazine ethane sulfonic acid
CNH: Cameroon National Herbarium
RPMI: Roswell Park Memorial Institute
SERM: Selective estrogen receptor modulators
SEM: Standard error of mean
SRB: Sulforhodamine-B
OVX: Bilaterally ovariectomized rats.
